# KHSRP knockdown inhibits papillary renal cell carcinoma progression and sensitizes to gemcitabine

**DOI:** 10.3389/fphar.2024.1446920

**Published:** 2024-10-08

**Authors:** Wei Song, Heng Zhang, Yi Lu, Houliang Zhang, Jinliang Ni, Lan Chang, Yongzhe Gu, Guangchun Wang, Tianyuan Xu, Zonglin Wu, Keyi Wang

**Affiliations:** ^1^ Department of Urology, Shanghai Shidong Hospital of Yangpu District, Shanghai, China; ^2^ Department of Urology, Shanghai Putuo District People’s Hospital, Tongji University, Shanghai, China; ^3^ Department of Urology, Shanghai 10th People’s Hospital, School of Medicine, Tongji University, Shanghai, China; ^4^ Guiqian International General Hospital, Guiyang, China; ^5^ Shanghai Putuo District Health Affairs Management Center, Hospital Operation Department, Shanghai, China; ^6^ Department of Neurology, Shanghai 10th People’s Hospital, School of Medicine, Tongji University, Shanghai, China

**Keywords:** papillary renal cell carcinoma, metastasis, KHSRP, gemcitabine, target therapy

## Abstract

Patients diagnosed with papillary renal cell carcinoma (pRCC) exhibit a high rate of clinical metastasis; however, the underlying molecular mechanism is unclear. In this study, KH-type splicing regulatory protein (KHSRP) participated in pRCC progression and was associated with metastasis. It was positively correlated with the hallmark of epithelial-mesenchymal transition. KHSRP inhibition effectively alleviated the cellular function of migration and invasion. Additionally, KHSRP knockdown inhibited the proliferative ability of pRCC cells. A pharmaceutical screening was based on the KHSRP protein structure. Gemcitabine (Gem) decreased KHSRP expression. UIO-66@Gem@si-KHSRP (UGS) nanoparticles (NPs) were prepared for targeted delivery and applied in both *in vitro* and *in vivo* experiments to explore the clinical transition of KHSRP. UGS NPs exhibited better performance in inhibiting cellular proliferation, migration, and invasion than Gem. Additionally, the *in vivo* experiment results confirmed their therapeutic effects in inhibiting tumor metastasis with excellent biosafety. The silico analysis indicated that KHSRP knockdown increased cytotoxic cell infiltration in the tumor microenvironment to potentiate anti-tumor effects. Thus, KHSRP can promote pRCC progression as an oncogene and serve as a target in clinical transition through UGS NP-based therapy.

## Introduction

Renal cell carcinoma (RCC) is a common tumor of the urinary system. RCC incidence is associated with the region and sex, with a higher incidence in men than in women and in urban than in rural areas. The age of onset ranges between 50 and 70 years (Mao et al., 2022). Papillary renal cell carcinoma (pRCC) is the most common pathological type of RCC apart from clear cell renal cell carcinoma (ccRCC). It accounts for 10%–15% of all RCC cases (Patard et al., 2005). pRCC is of two types, namely, types 1 and 2. Type 1 is more common and develops gradually. Type 2 is more malignant and develops rapidly (Peruzzi and Bottaro, 2006). p pRCC can invade, compress, and destroy the renal calyx and pelvis. Simultaneously, it can break through the outer renal peritoneum to form a vascular thrombus or metastasize to the lymph nodes (LN) and other organs. LN is the most common site of pRCC metastasis (Dudani et al., 2021; Karaman and Detmar, 2014). However, the biological characteristics of pRCC are unclear.

KH-type splicing regulatory protein (KHSRP) is a multifunctional RNA-binding protein involved in the transcriptional and post-transcriptional regulation of gene expression (Palzer et al., 2022; Trabucchi et al., 2009). KHSRP is central to numerous biological processes, including innate and adaptive immune responses, DNA damage response, inflammatory diseases, tissue remodeling, and lipid metabolism (Gherzi et al., 2014). It may play opposing roles at different stages of cancer development. For example, KHSRP inhibits motility in brain tumors and non-small cell lung cancer (NSCLC); furthermore, it is associated with a good prognosis (Yang et al., 2013; Fujita et al., 2017). In NSCLC, the anti-metastatic effects of KHSRP have been associated with inducing microRNA (miR)-23a maturation, which mediates early growth response gene 3 (EGR3) mRNA degradation. However, KHSRP can promote the growth or invasion of oesophageal squamous cell carcinoma and melanoma by enhancing miRNA maturation, such as miR-21, miR-130b, and miR-301, and by inducing Killin mRNA destabilization (Liu et al., 2019). Despite its roles in different cancers, the association between KHSRP and pRCC, including its function, molecular mechanism, and clinical potential, remains unclear.

In this study, we identified a positive correlation between KHSRP and epithelial-mesenchymal transition markers through a bioinformatics analysis. Subsequent cell function experiments demonstrated that KHSRP inhibition effectively alleviated the proliferation, migration, and invasion functions of pRCC cells. Drug screening results indicated that gemcitabine (Gem) targeted KHSRP and reduced its expression. Additionally, UIO-66@Gem@si-KHSRP (UGS) nanoparticles (NPs) were prepared for targeted delivery and applied in *ex vivo* experiments. UGS NPs exhibited superior efficacy in inhibiting cell proliferation, migration, and invasion, compared with Gem. Thus, KHSRP, an oncogene, is central to pRCC progression and can be considered a promising target for clinical translation by UGS NP-based therapies.

## Methods and materials

### Data collection and bioinformatics analysis

pRCC clinical data were obtained from The Cancer Genome Atlas (TCGA) database (https://genome-cancer.ucsc.edu/). A total of 291 pRCC and 32 normal tissue transcriptome data with the RNA-seq count type were analyzed to compare the clinical features. All normalized data were analyzed through the R software. The associations between KHSRP expression and clinical features, including the tumor (T), nodes (N), metastases (M) and pathological stages, were determined. Additionally, the predictive performances of KHSRP expression towards patient survival were evaluated through the Kaplan–Meier curves. Moreover, the Kyoto Encyclopedia of Genes and Genomes (KEGG), Gene Ontology (GO), and Gene Set Enrichment Analysis data were analyzed using the clusterProfiler package (R software).

### Cell culture and transfection

CAKI-2 and ACHN pRCC cell lines were brought from the Cell Bank of the Chinese Academy of Sciences (Shanghai, China). These cell lines were cultured in Minimum Essential Medium and McCoy’s 5A media (Gibco, United States) added with 10% fetal bovine serum (FBS, Hyclone, United States) and 1% penicillin/streptomycin (P/S, YEASEN, China), respectively, at 37°C in 5% CO_2_. Small interfering KHSRP RNAs (si-KHSRP) were obtained from RIBOBIO (China) and transfected in the cells through jetPRIME (YEASEN, China). The si-KHSRP sequence was GCGTGCGGATACAGTTCAA. For inducible gene silencing, cells were cultured in 6-wells palate for 24 h with the confluence around 60%. Next, the cells were treated with si-KHSRP (5 µL per well) for 24–48 h in the presence of Lipofectamine 3000 Reagent. The cells were collected for the next experiments after the confirmation of KHSRP knockdown with the results of WB and qPCR.

### RNA extraction and quantitative real-time PCR

Total RNA was extracted using the Trizol reagent (Invitrogen, United States), with the RNA concentration measured using a Nanodrop 2000 spectrophotometer (Thermo Scientific, United States). After complementary DNA synthesis, KHSRP expression was detected by reverse transcription-quantitative polymerase chain reaction with KHSRP-specific primers (Sangon Biotech, Shanghai, China). Glyceraldehyde-3-phosphate dehydrogenase (GAPDH) was considered as a control to calculate the relative RNA expression. Supplementary Material enlists the primer sequences (Supplementary Table S1).

### Western blotting

The tissues or cells were lysed on ice for 30 min using a lysis buffer (PC102, Epizyme, Shanghai, China). The protein concentration was determined using a bicinchoninic acid protein assay kit (ZJ101, Epizyme, Shanghai, China). The proteins (20 μg) were separated by sodium dodecyl sulfate-polyacrylamide gel electrophoresis on 10% polyacrylamide gels and transferred onto nitrocellulose membranes (WJ004, EpiZyme, Shanghai, China). These membranes were blocked with 5% skim milk at room temperature for 1 h, followed by overnight incubation with primary antibodies at 4°C. After thorough washing, they were incubated with horseradish peroxidase-conjugated secondary antibodies at room temperature for 1 h, followed by membrane blotting using enhanced chemiluminescence detection reagents (NCM, Suzhou, China). Chemiluminescence signals were detected using an imaging system (AI600, GE, United States). Individual protein band intensities were measured using ImageJ software (NIH, Rasband, WS, United States). KHSRP and GAPDH antibodies were obtained from Abcam.

### Wound healing

Wound healing experiments were conducted to assess the migration ability of cells. First, the cells underwent different treatments. Second, after 12 h of transfection, they were digested into cell suspension and seeded into six-well plates. The cell fusion rate reached 90%; subsequently, the tip of the pipette gun was used to scratch the cell layer. The tip was maintained vertically. Third, after scratching, the cells were washed with phosphate-buffered saline (PBS) three times to remove the shed cells and added to the 1640 culture medium. Finally, a similar field of view was selected for photography at 0 h and 24 h time points. The wound healing rate was determined through the ImageJ software.

### Transwell assay

The invasion and migration abilities were measured through a transwell assay. First, the cells underwent different treatments. Second, after 12 h of transfection, they were digested into a cell suspension and numbered for the ensuing step. For the transwell assay, 5^104 cells in 200 μL of serum-free medium were cultured in the upper chambers, with (invasion) or without (migration) Matrigel. Additionally, 500 μL of medium with 10% FBS was loaded in the lower chambers. Third, the cells were incubated in 5% CO_2_ and 37°C for 24 h. After incubation, the chamber was removed and the remaining cells were extracted. Finally, the upper surface of the upper chamber was gently rubbed with a cotton swab. The lower surface of the lower chamber was washed with PBS and fixed and stained for photography.

### 5-ethynyl-2 deoxyuridine analysis for cell proliferation

The cells were cultured in 24-well plates after treatment and exposed to 10 μM 5-ethynyl-2 deoxyuridine (EdU) (EpiZyme, China) for 2 h at 37°C in 5% CO_2_. Pre-treated cells were fixed with 4% paraformaldehyde for the ensuing stage. After washing with PBS/0.3% bovine serum albumin, they were incubated with Alexa Fluor 555 and DAPI in the dark. The EdU results were visualized through the Leica DM6 B upright microscope system (Leica, Germany).

### Colony formation

A colony formation assay was performed to evaluate cell proliferation. First, the cells underwent different treatments. Second, after 12 h of transfection, they were digested into a cell suspension and numbered for the subsequent step. According to a density gradient of 1,000 cells per well, they were inoculated into a six-well plate. Third, each well was filled with the complete culture medium up to 2,000 μL. The cells were incubated in 5% CO_2_ and 37°C for 14 days. Fourth, the culture was terminated upon observing visible clones in the culture dish. The culture medium was discarded and carefully soaked twice in PBS. Finally, the colonies were fixed and stained for photography after air drying. The number of clones was directly quantified.

### Pharmaceutical screening for KHSRP

A pharmaceutical screening was based on the KHSRP protein crystal structure. KHSRP protein structure was obtained from the Protein Data Bank database. The structure was processed using UCSF Chimera, and the SiteMap database was used to predict the optimal binding sites. The screening library comprised the drug molecules approved for marketing by the U.S. Food and Drug Administration. It was derived from the ZINC20 database, a specialized screening molecule library consisting of 1,766 drug molecules.

### USG NPs preparation and characterization

UIO-66 NPs were prepared for targeted delivery (Zhou et al., 2023; Jarai et al., 2020). Briefly, 90.0 mg (0.386 mmol) of ZrCl4 and terephthalic acid were separately added to N, N-dimethylformamide (DMF) in a 20 mL vial and sonicated for dissolution. Upon obtaining a clear solution, the samples were heated at 110°C for 24 h. After heating, they were diluted in DMF for 72 h and washed in methanol. Finally, they were dissolved in diethylpyrocarbonate (DEPC)-treated water. The size of UIO-66 NPs was measured through digital light synthesis. UGS NPs (20 µL) were dissolved in distilled water (1 mL). And the size of the UGS NPs were measured through a particle size potentiometer (Nano ZS90, Worcestershire, United Kingdom). Gem (100 μg/mL) was added to the water-diluted UIO-66 NPs and stirred for 24 h to obtain the UIO-66@Gem NPs. Then, UIO-66@Gem NPs were collected after centrifugation and washed three times. Varying concentrations of UIO-66@Gem NPs and si-KHSRP were dissolved in DEPC-treated water to prepare the UGS NPs through electrostatic interaction. Gem release was assessed through absorbance changes at 280 nm using the ultraviolet-visible-near infrared spectrometer. The experiments were measured through a UV-vis spectrophotometer. Gel electrophoresis was conducted to detect si-KHSRP encapsulation through UGS NPs. The gels were prepared and the procedures were conducted according to the previously reports (Mao et al., 2022). Using the empty plasmid as a control, UIO-66@Gem NPs with the different concentration were examined to evaluate the RNA encapsulation capability. To examine the cellular internalization of UGS NPs, the tumor cells were treated with UGS NPs for 12 h. Then, the cells were digested for the bio-TEM. The cells were collected through glutaraldehyde fixative (2.5%) overnight under 4°C. Next, the prepared cells were washed and dehydrated for the polymerization in spurr’s low-viscosity solution at 60°C. Finally, the cells visualized through bio-TEM.

### Animal models

A lung metastasis model was established in 4-week-old female BALB/c nude mice (Charles River, China). Briefly, 1 × 105 cells were injected into the bloodstream via the tail vein. After 3 weeks, the mice were intraperitoneally injected with D-luciferin (Goldbio, United States) (100 mg/kg), and images were captured using the AniView100 imaging system (Guangzhou, China). In the UGS NP treatment model, the mice were intravenously injected with PBS and NPs every 3 days (200 μL).

### Statistical analysis

All analyses were performed using R (v.3.6.3). A Wilcoxon test or Student’s t-test was conducted to investigate the association between clinical features and KHSRP expression. Kaplan-Meier analysis was conducted to evaluate the TCGA patient survival rates. A *p*-value <0.05 indicated statistical significance. All data are presented as mean ± SD, and significant differences were determined based on the *t*-test results.

## Results

### KHSRP expression is negatively associated with pRCC clinical features

**FIGURE 1:**
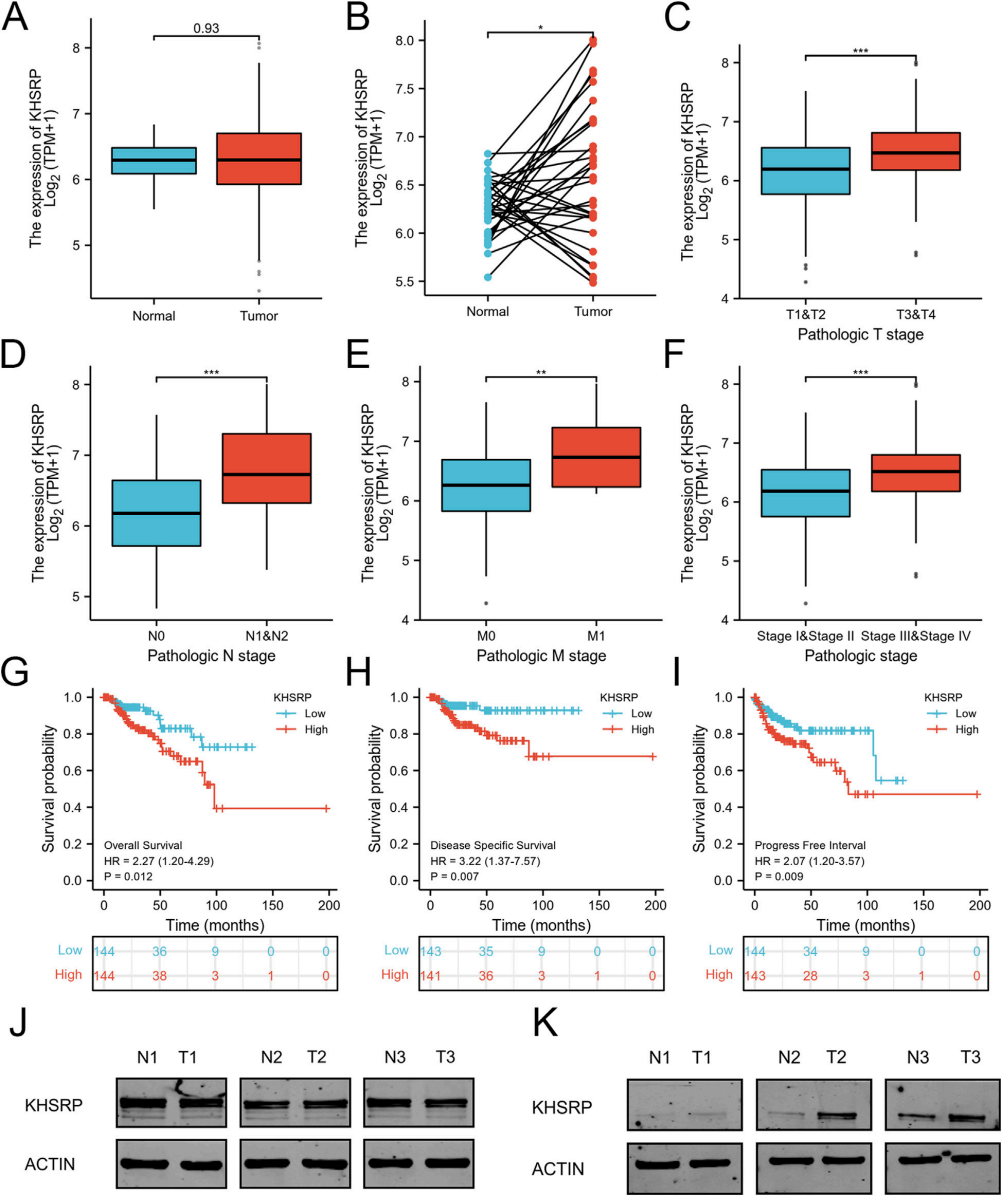
Expression analysis of KHSRP in pRCC. **(A)** Expression differences of KHSRP in the matched analysis. **(B)** Expression of KHSRP in ccRCC tissues and adjacent normal tissues. **(C–F)** Relationship between the expression of KHSPR and clinicopathological features in pRCC. **(G–I)** Kaplan-Meier curves analysis of KHSRP expression in predicting overall survival, disease-specific survival, and progress free interval. **(J)** KHSRP protein expression in ccRCC paired samples. **(K)** KHSRP protein expression in pRCC paired samples.

KHSRP participated in tumor progression as an oncogene. KHSRP expression was elevated in the tumor tissues based on the matched analysis (*p* < 0.05; Figure 1B), but not the comparative analysis (Figure 1A). Meanwhile, the relationship between KHSRP expression and T-stage (*p* < 0.05; Figure 1C), N-stage (*p* < 0.05; Figure 1D), M-stage (*p* < 0.05; Figure 1E), and pathological stage (*p* < 0.05; Figure 1F). The detailed information of all samples was exhibited in Table 1. Additionally, the receiver operating characteristic curve analysis evaluated the predictive performances of KHSRP expression in determining the higher TNM and pathological stages (Supplementary Figure S1A). Results of the Kaplan-Meier analysis confirmed that high KHSRP expression was associated with poor overall survival (*p* = 0.012; Figure 1G), disease specific survival (*p* = 0.007; Figure 1H), and progression free interval (*p* = 0.009; Figure 1I). All the results confirmed that KHSRP as the oncogene, participated in the pRCC tumor progression. Finally, protein levels in ccRCC and pRCC clinical samples were examined, and it was demonstrated that KHSRP was not differentially expressed in paired ccRCC samples (Figure 1J) and was highly expressed in pRCC (Figure 1K).

**TABLE 1:** The detailed information of clinical samples.

Characteristics	Low expression of KHSRP	High expression of KHSRP	P value
n	145	146	
Pathologic T stage, n (%)			< 0.001
T1&T2	125 (43.3%)	102 (35.3%)	
T3&T4	19 (6.6%)	43 (14.9%)	
Pathologic N stage, n (%)			0.005
N0	27 (34.6%)	23 (29.5%)	
N2&N1	6 (7.7%)	22 (28.2%)	
Pathologic M stage, n (%)			0.736
M0	43 (41.3%)	52 (50%)	
M1	3 (2.9%)	6 (5.8%)	
Pathologic stage, n (%)			0.001
Stage I&Stage II	106 (40.6%)	88 (33.7%)	
Stage III&Stage IV	21 (8%)	46 (17.6%)	
OS event, n (%)			0.010
Alive	131 (45%)	116 (39.9%)	
Dead	14 (4.8%)	30 (10.3%)	
DSS event, n (%)			0.005
No	137 (47.7%)	122 (42.5%)	
Yes	7 (2.4%)	21 (7.3%)	
PFI event, n (%)			0.031
No	123 (42.3%)	109 (37.5%)	
Yes	22 (7.6%)	37 (12.7%)	

## Inhibition of KHSRP alleviate the progression of pRCC

**FIGURE 2:**
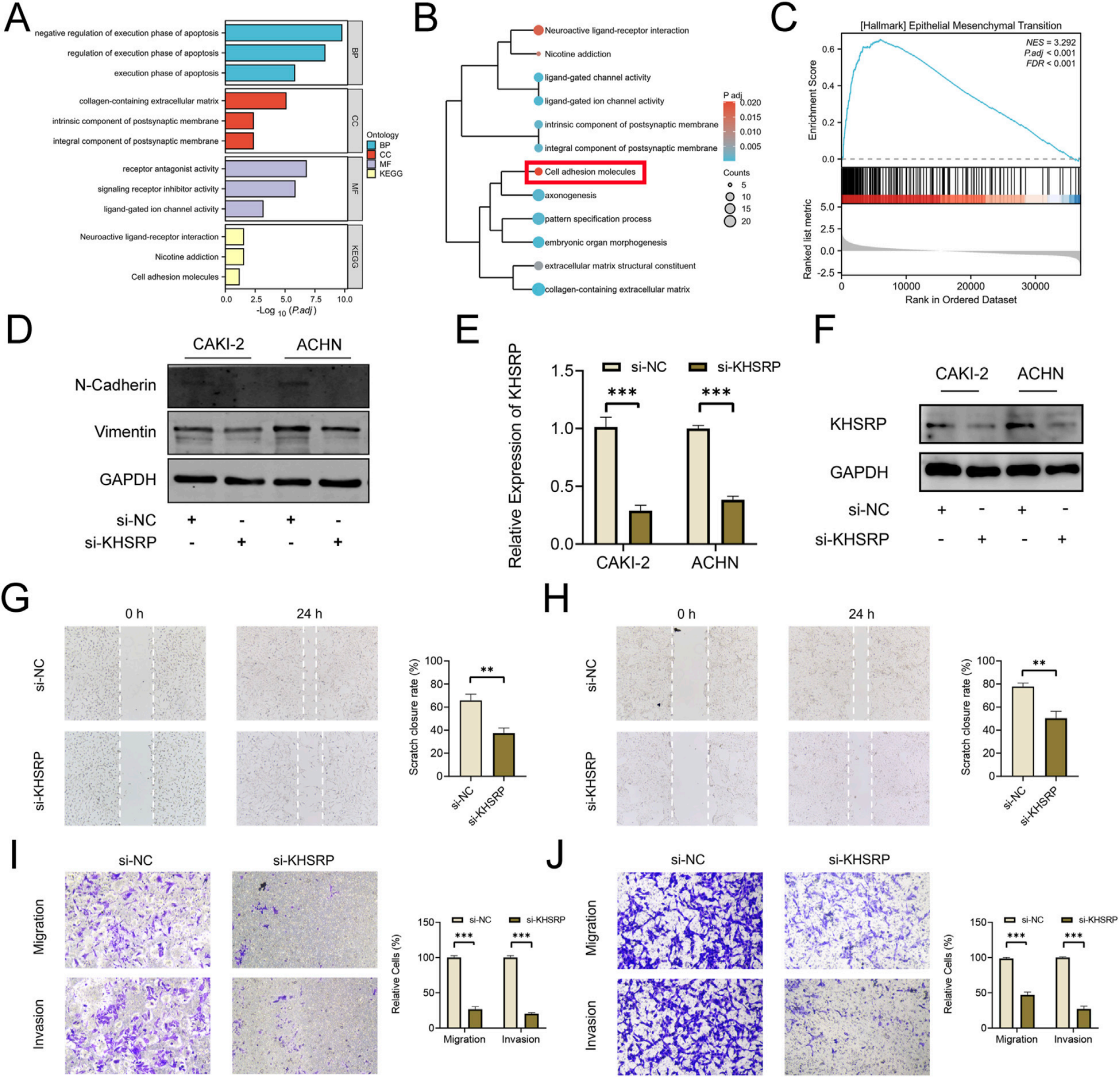
KHSRP knockdown inhibits cell migration and invasion *in vitro*. **(A)** GO and KEGG analysis of KHSRP related functional genes in pRCC. **(B)** GO and KEGG analysis of KHSRP positively related functional genes in pRCC. **(C)** GSEA analysis of KHSRP related functional genes in pRCC. **(D)** Western blotting analysis of EMT marker expression in 2 cell lines after transfection. **(E, F)** qPCR and Western blot analysis of KHSRP expression in 2 cell lines after transfection. **(G, H)** Wound-healing assay results after KHSRP knockdown in 2 cell lines for migration measurements. **(I, J)** Transwell assay results for cell migration and invasion after KHSRP knockdown in 2 cell lines.

**FIGURE 3:**
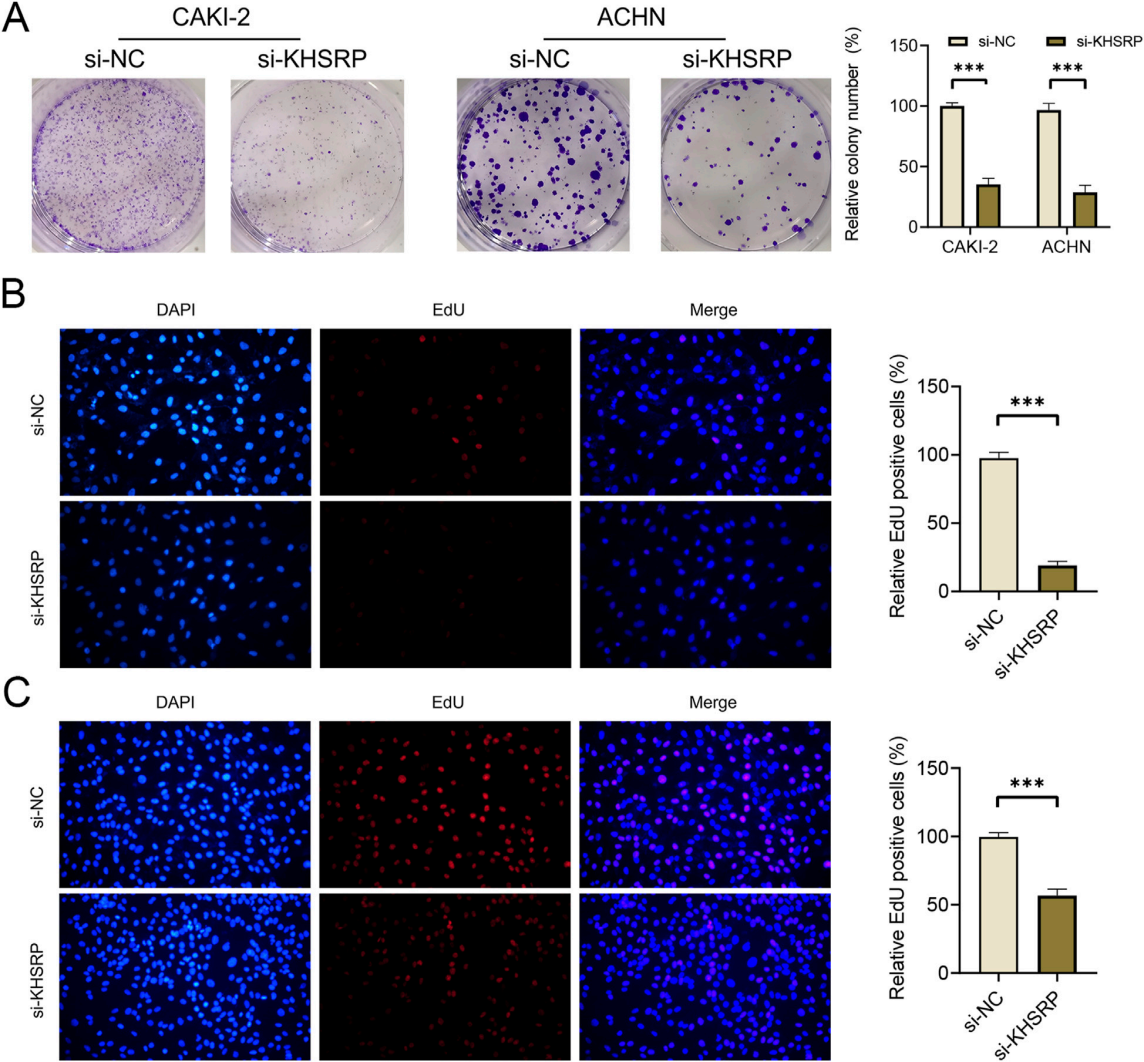
KHSRP knockdown inhibits cell proliferation *in vitro*. **(A)** Results of KHSRP knockdown in 2 cell lines evaluated through the colony assay. **(B, C)** EdU results of KHSRP knockdown in cell proliferation with the quantitative analysis in the right.

To explore the potential function of KHSRP, the correlated functional genes were screened and analyzed. GO and KEGG analyses were based on the screened functional genes. KHSRP was associated with the negative regulation of the execution phase of apoptosis according to the biological process analysis (Figure 2A). The correlated functional genes were divided into positive and negative related subgroups, with separate GO and KEGG analyses (Figure 2B; Supplementary Figure 1B). The KEGG analysis confirmed that KHSRP was positively correlated with the cell adhesion molecules (Figure 2B). Meanwhile, the GESA analysis confirmed that KHSRP was positively associated with the hallmark of epithelial-mesenchymal transition (Figure 2C). Additionally, Western blot analysis demonstrated that KHRSP knockdown reduced the expression of epithelial-mesenchymal transition markers (Figure 2D). Based on the silico results, the function of KHSRP in promoting pRCC progression was explored *in vitro*. CAKI-2 and ACHN exhibited higher KHSRP expression than HK-2 (Supplementary Figures S1C, S2A). si-KHSRP treatment effectively inhibited KHSRP mRNA and protein expressions (Figures 2E,F). Wound healing and transwell assays were conducted with si-KHSRP treatment to assess KHSRP function in promoting pRCC progression. KHSRP knockdown alleviated the cellular migration and invasion in both cell lines (Figures 2G–J). Moreover, pRCC cell line proliferation was evaluated before/after si-KHSRP treatment. KHSRP inhibition suppressed the proliferative ability of pRCC cells (Figures 3A–C).

### Pharmaceutical screening targets KHSRP

**FIGURE 4:**
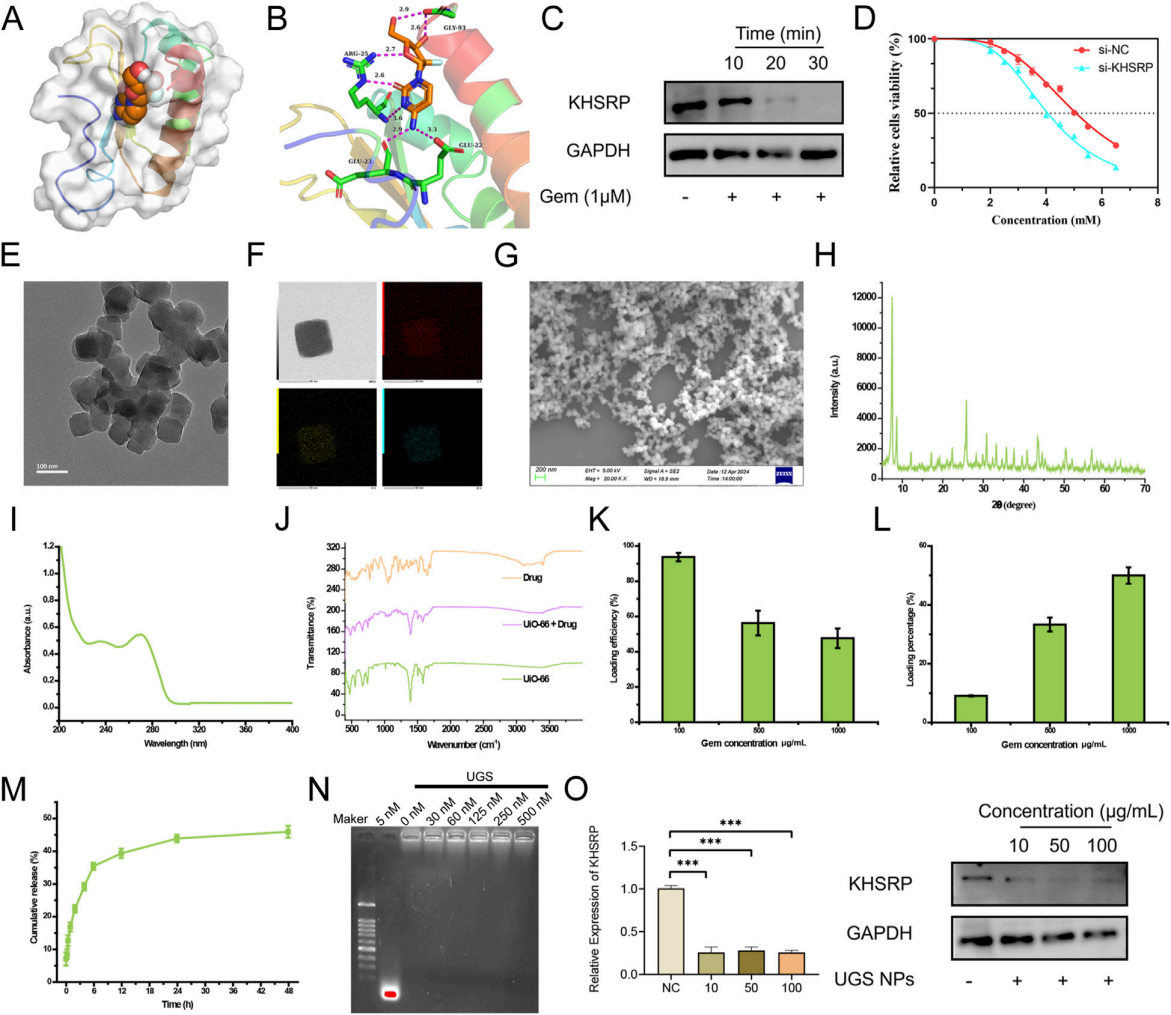
Pharmaceutical screening for KHSRP. **(A, B)** Gem could bind to the protein structure of KHSRP with 7 hydrogen bonds formed. **(C)** Western blot analysis of KHSRP expression in ACHN after Gem treatments. **(D)** IC50 for Gem in normal and low expression groups. **(E)** TEM image of UIO-66. **(F)** EDS mapping image of UIO-66. **(G)** SEM image of UIO-66. **(H)** XRD results of UIO-66. **(I)** FTIR results of UIO-66, Gem, and UIO-66@Gem. **(J)** UV-absorbance of Gem. **(K, L)** Gem loading efficiency and percentage through UIO-66. **(M)** UIO-66 could release Gem. **(N)** si-KHSRP could be loaded through UGS. **(O)** qPCR and Western blot analysis of KHSRP expression in ACHN after the treatment of UGS.

To explore the potential clinical therapy based on KHSRP, pharmaceutical screening was conducted to investigate the targeted drugs. Gem could bind to the KHSRP protein structure as a chemotherapy drug (Figure 4A). Gem and KHSRP formed seven hydrogen bonds, with a docking fraction of −5.769 kcal/mol (Figure 4B; Supplementary Figure S1D). Additionally, Gem treatments effectively decreased KHSRP expression in a time-dependent manner (Figure 4C), indicating Gem inhibited KHSRP expression. Furthermore, we investigated the impact of KHSRP knockdown on the IC50 of cells to Gem. Our findings revealed a notable decline in IC50 following KHSRP knockdown (Figure 4D), suggesting that KHSRP depletion enhanced the sensitivity of cells to Gem. UIO-66 NPs were prepared for the targeting delivery of Gem. It was reported that UIO-66 NPs were in an advantageous range for cellular uptake, and contained the advantage for drug delivery (Jarai et al., 2020). Both transmission electron microscopy and scanning electron microscopy confirmed the successful preparation of UIO66 NPs with uniform size and morphology (Figures 4E,G). The size of UIO66 NPs was measured through the digital light synthesis, which was around 50 nm (Supplementary Figure S2B). The energy dispersive x-ray spectroscopy mapping results affirmed that UIO-66 NPs comprised C, O, and Zr elements (Figure 4F). Meanwhile, the X-ray diffraction results suggested that the prepared UIO-66 NPs possessed the identical (111) crystal plane of 7.48o and (002) 8.62o as reported previously (Zhou et al., 2023) (Figure 4H). UIO-66@Gem NPs were synthesized (Wu et al., 2019). Fourier transform infrared spectroscopy results indicated that the UiO-66 NPs were loaded with Gem, matching both characteristic peaks (Figure 4I). Additionally, the Gem loading and releasing assays were based on their absorbance (Figure 4J). UIO-66 NPs effectively loaded and subsequently released Gem (Figures 4K–M). Considering the anti-tumor function of si-KHSRP, UGS NPs were prepared to achieve the combination therapy effect. UGS NPs effectively loaded the si-KHSRP at a concentration of 500 nM (Figure 4N). *In vitro* experiments were conducted to examine their anti-tumor performances. It was confirmed that UGS NPs could been effectively uptake by the tumor cells (Supplementary Figure S2C). UGS effectively inhibited KHSRP expression in both pRCC cell lines (Supplementary Figure S2D). Meanwhile, these inhibiting performances were exhibited in a concentration-dependent manner under UGS NP treatment (Figure 4O).

### UGS NPs inhibit tumor progression for potential clinical therapy

**FIGURE 5:**
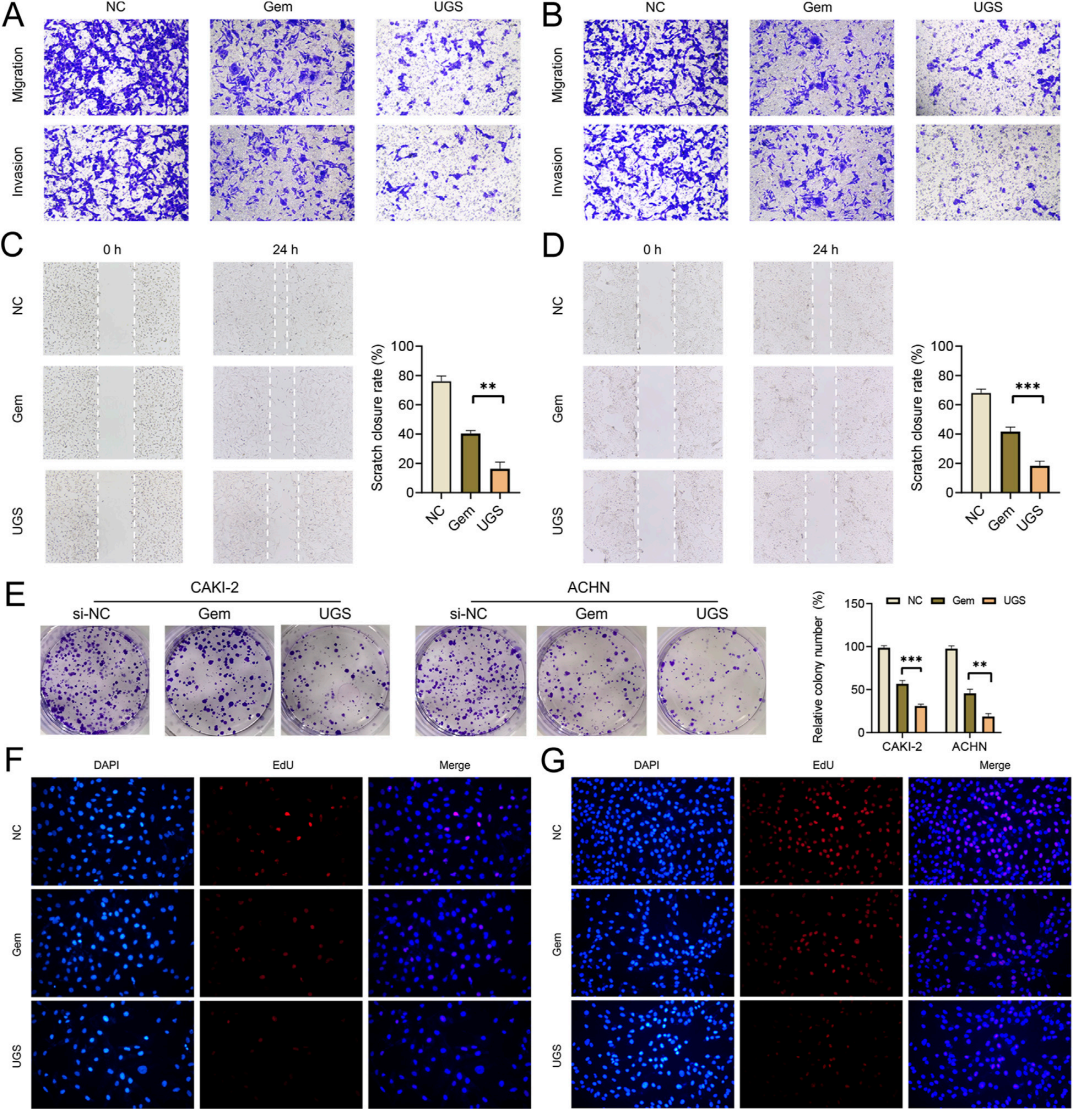
UGS treatment inhibits cell proliferation, migration, and invasion *in vitro*. **(A, B)** Transwell assay results for cell migration and invasion after different treatments in 2 cell lines. **(C, D)** Wound-healing assay results after different treatments in 2 cell lines for migration measurements. **(E)** Results of different treatments towards proliferation in 2 cell lines evaluated through the colony assay. **(F, G)** EdU results of different treatments in cell proliferation.

**FIGURE 6:**
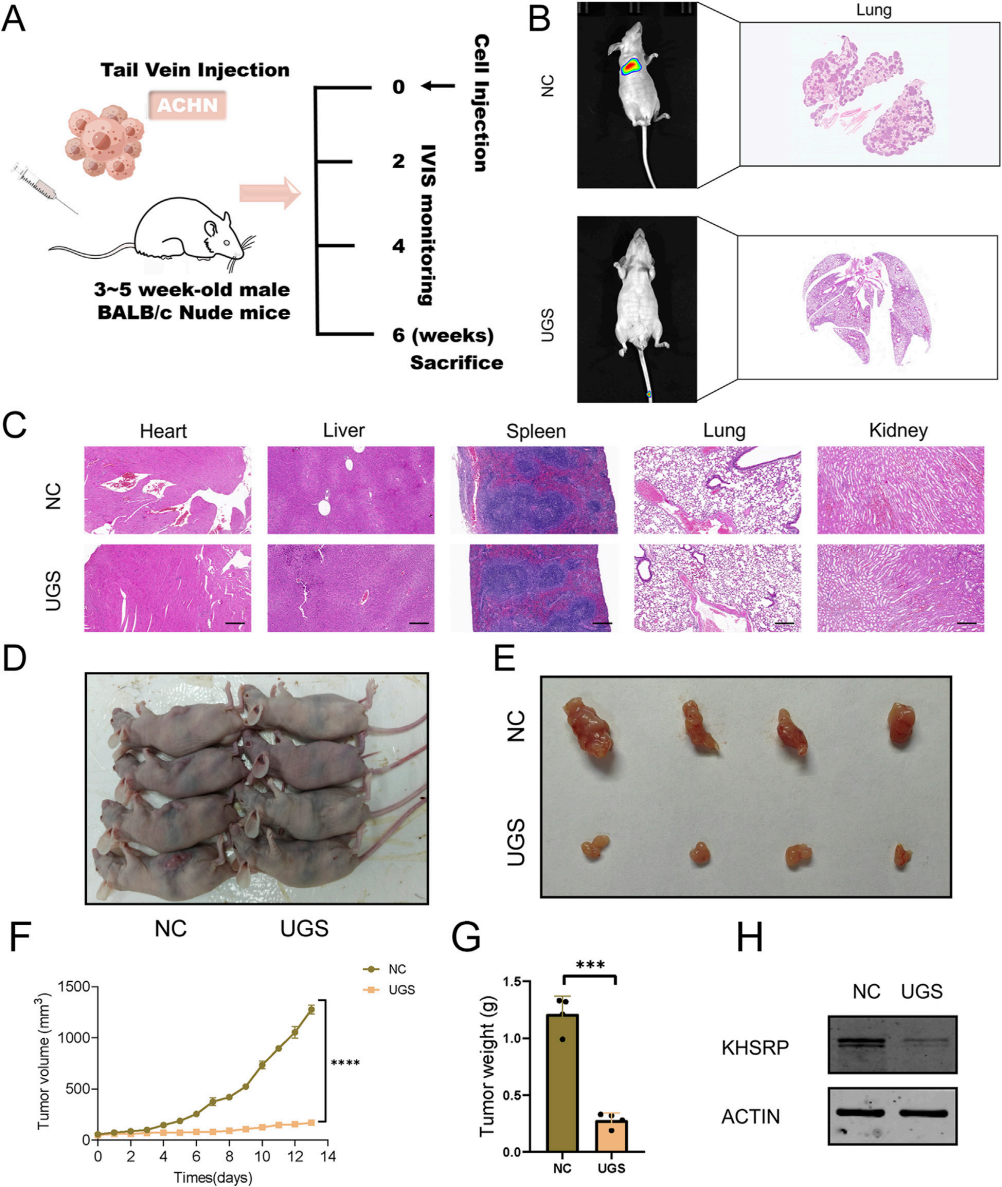
UGS treatment suppresses tumor metastasis *in vivo*. **(A)** Schematic diagram of animal experiment process. **(B)** IVIS and HE images of tumors in the two subgroups. **(C)** HE images of main organs in the two subgroups. **(D–E)** Primary tumour images of two subgroups. **(F)** Subcutaneous tumour growth curve. **(G)** Weight statistics of subcutaneous tumours. **(H)** KHSRP protein expression in subcutaneous tumours.

To measure the anti-tumor function of UGS NPs, wound healing, and transwell assays were conducted. UGS NP treatments strongly alleviated the cellular migration and invasion in both cell lines, compared with Gem (Figures 5A–D and Supplementary Figures 2E, F). Furthermore, better pRCC cell line proliferation was observed before/after UGS NP treatment, compared with Gem treatment. The experimental evidence confirmed that UGS NP treatment suppressed the proliferative ability of pRCC cells (Figures 5E–G and Supplementary Figures 2G, H). All *in vitro* experiment results confirmed the anti-tumor function of UGS NPs. UGS NP function was explored *in vivo* using a lung metastasis model (Figure 6A). The *in vivo* imaging system confirmed that UGS NPs significantly inhibited lung metastasis in nude mice, compared with the negative control (Figure 6B). Additionally, histogram equalization results of the primary organs of UGS-treated mice affirmed that UGS NPs possessed excellent biosafety, causing no noticeable damage (Figure 6C). Meanwhile, the blood tests were conducted to evaluate the biosafety of UGS NPs, which indicting that UGS treatments caused no obviously damages to the red blood cells, hemoglobin, platelets, and white blood cells (Supplementary Figures S2I–K). We finally constructed an *in situ* model of pRCC to explore the potential of UGS NPs to inhibit primary tumour growth. The findings demonstrated that UGS NPs markedly suppressed the proliferation of subcutaneous tumours in comparison to the control group (Figures 6D–G). Additionally, the KHSRP protein level in subcutaneous tumours in the UGS NPs group was also found to be significantly reduced (Figure 6H).

## Discussion

pRCC prognosis is more favorable than ccRCC prognosis, which is confined to the organs. However, the histological prognosis of pRCC in metastatic disease is less favorable than that of ccRCC (Steffens et al., 2012). Researchers have made advances in pRCC treatment, including combination strategies with targeted therapies and immune checkpoint inhibitors. However, its overall progress lags behind that of ccRCC, partly because of the heterogeneity of different pRCC subtypes (Chawla et al., 2023). In this study, *ex vivo* and *in vivo* experiments demonstrated that UGS NPs effectively inhibited the proliferation, migration, and invasion of pRCC cells. *In vivo* experiments demonstrated the outstanding therapeutic effects of UGS NPs in suppressing lung metastasis. Furthermore, KHSRP knockdown increased the infiltration of cytotoxic cells within the tumor microenvironment, thereby enhancing the anti-tumor effect.

KHSRP is a multifunctional nucleic acid-binding protein comprising 711 amino acids. It comprises a terminal structural domain of amino acids, a central structural domain of four KH motifs, and a terminal structural domain of carboxyl groups (Nicastro et al., 2012). The KH1 and KH4 domains of the central structure can interact with other proteins to form a β-folded structure, whereas the KH2 and KH3 domains comprise negative regulatory binding sites (Yan et al., 2019). Furthermore, KHSRP can interact with other proteins (Diaz-Moreno et al., 2009) and bind to not only ribonucleic acids but also other proteins. Additionally, it plays regulatory roles (Gherzi et al., 2010). Furthermore, KHSRP is involved in the pathophysiological regulation of neuromuscular disorders (Amirouche et al., 2013), obesity (Lin et al., 2014), type II diabetes mellitus (Briata et al., 2016), and cancer (Yuan et al., 2017).

To date, most studies have focused on the function and potential mechanisms underlying KHSRP in tumorigenesis and development. However, KHSRP plays distinct roles in different tumors. Chien et al. (Chien et al., 2017) demonstrated that KHSRP is associated with favorable survival and prognosis in patients with non-small cell lung cancer. KHSRP inhibits the invasion and migration of non-small cell lung cancer through the miR-23a/EGR3 axis. However, Bikkavilli et al. (Bikkavilli et al., 2017) reported that KHSRP silencing attenuates the malignant biological behavior of cell proliferation, migration, and invasion, suggesting its oncogenic role in lung cancer. Furthermore, Wang et al. (2016) demonstrated that KHSRP facilitates the cell cycle and enhances chemoresistance to adriamycin in breast cancer. In conclusion, the diverse functions of KHSRP in different tumors indicate that its role in cancer progression and drug sensitivity is highly dependent on the tumor cell type. To the best of our knowledge, this is the first study to elucidate the role of KHSRP in pRCC cells. KHSRP knockdown inhibits the malignant biological behavior of pRCC during clinical treatment.

Cancer treatment has witnessed substantial advances in the long-term evolution of medicine. In addition to the advancement and development of surgical approaches, neoadjuvant therapy has promoted longevity, based on drug development and innovation. For instance, preoperative and postoperative radiotherapy and chemotherapy have become the mainstay of cancer treatments, relying on the high drug toxicity to cancer cells as well as high killing efficiency. However, conventional chemotherapeutic agents have certain limitations, including non-targeted distribution and poor solubility *in vivo*, poor bioavailability, and rapid blood clearance (Cho et al., 2008; Han et al., 2013). The development of nanomaterials has led to their use in tumor diagnosis and treatment. This can be attributed to their good biocompatibility, high drug-carrying efficacy, controllable drug-release ability, and enhanced tumor penetration (Qin et al., 2017; Zhu et al., 2023). Adjuvant nanocarriers are used to address these issues, such as inorganic nano frames for drug delivery (Zhang et al., 2013; Tarn et al., 2013). These nanocarriers protect the drug from rapid metabolism or clearance by the blood, liver, and kidneys. Additionally, they facilitate long-term drug accumulation within solid tumors through enhanced permeability and retention effects (Kobayashi et al., 2013; Maeda, 2015). UiO-66 is a zirconium-based metal-organic framework (MOF) consisting of a biocompatible and water-stable terephthalic acid ligand, rendering it an optimal material for drug delivery applications (Orellana-Tavra et al., 2015). UiO-66 has been utilized in numerous drug delivery applications, including oral, dermal, and intravenous drug delivery (Javanbakht et al., 2019). Despite the extensive range of delivery methods utilizing UiO-66 and other MOFs, few studies have used formulated UiO-66 to deliver Gem for pRCC treatment.

KHSRP knockdown inhibits the malignant biological behavior of pRCC, and Gem can reduce KHSRP expression. UGS NPs were constructed for the targeted delivery of Gem and applied in *ex vivo* experiments. UGS NPs exhibited superior efficacy in inhibiting pRCC proliferation, migration, and invasion. Additionally, they exhibited a favorable biosafety profile for pRCC treatment, compared with Gem alone. Thus, KHSRP is central to pRCC progression and can be considered a potential target for clinical translation through UGS NP-based therapies.

## Data Availability

The original contributions presented in the study are included in the article/Supplementary Material, further inquiries can be directed to the corresponding authors.

## Supplementary material

SUPPLEMENTARY FIGURE S1 Clinical and silico analysis of KHSRP. **(A)** ROC curves of KHSRP expression in predicting TNM-stage and pathological stage. **(B)** GO and KEGG analysis of KHSRP negatively related functional genes in pRCC. **(C)** KHSRP expression in pRCC cell lines. **(D)** Gem could bind to the protein structure of KHSRP with 7 hydrogen bonds formed.

SUPPLEMENTARY FIGURE S2 UGS NPs inhibit KHSRP expression. **(A)** KHSRP expression in different cell lines. **(B)** Particle size distribution of UGS NPs. **(C)** Bio-TEM images for cell phagocytosis of UGS NPs. **(D)** Western blot analysis of KHSRP expression in 2 cell lines after the treatment of UGS. **(E, F)** Quantitative analysis of transwell assay results in the 2 cell lines. **(G, H)** Quantitative analysis of EdU assay results in the 2 cell lines. **(I–K)** Blood tests for red blood cells, hemoglobin, platelets, and white blood cells after UGS NPs treatments.
