# Supplementary material for: KHSRP knockdown inhibits papillary renal cell carcinoma progression and sensitizes to gemcitabine
Source: Front Pharmacol. 2024 Oct 8;15:1446920. doi: 10.3389/fphar.2024.1446920 (PMC11493689; doi:10.3389/fphar.2024.1446920)
Supplement: Supplementary file 1 [file Table1.docx]

| Characteristics | Low expression of KHSRP | High expression of KHSRP | P value |
| --- | --- | --- | --- |
| n | 145 | 146 |  |
| Pathologic T stage, n (%) |  |  | < 0.001 |
| T1&T2 | 125 (43.3%) | 102 (35.3%) |  |
| T3&T4 | 19 (6.6%) | 43 (14.9%) |  |
| Pathologic N stage, n (%) |  |  | 0.005 |
| N0 | 27 (34.6%) | 23 (29.5%) |  |
| N2&N1 | 6 (7.7%) | 22 (28.2%) |  |
| Pathologic M stage, n (%) |  |  | 0.736 |
| M0 | 43 (41.3%) | 52 (50%) |  |
| M1 | 3 (2.9%) | 6 (5.8%) |  |
| Pathologic stage, n (%) |  |  | 0.001 |
| Stage I&Stage II | 106 (40.6%) | 88 (33.7%) |  |
| Stage III&Stage IV | 21 (8%) | 46 (17.6%) |  |
| OS event, n (%) |  |  | 0.010 |
| Alive | 131 (45%) | 116 (39.9%) |  |
| Dead | 14 (4.8%) | 30 (10.3%) |  |
| DSS event, n (%) |  |  | 0.005 |
| No | 137 (47.7%) | 122 (42.5%) |  |
| Yes | 7 (2.4%) | 21 (7.3%) |  |
| PFI event, n (%) |  |  | 0.031 |
| No | 123 (42.3%) | 109 (37.5%) |  |
| Yes | 22 (7.6%) | 37 (12.7%) |  |

Table 1 The detailed information of clinical samples.
