# Supplementary figures and images for: KHSRP knockdown inhibits papillary renal cell carcinoma progression and sensitizes to gemcitabine
Source: Front Pharmacol. 2024 Oct 8;15:1446920. doi: 10.3389/fphar.2024.1446920 (PMC11493689; doi:10.3389/fphar.2024.1446920)

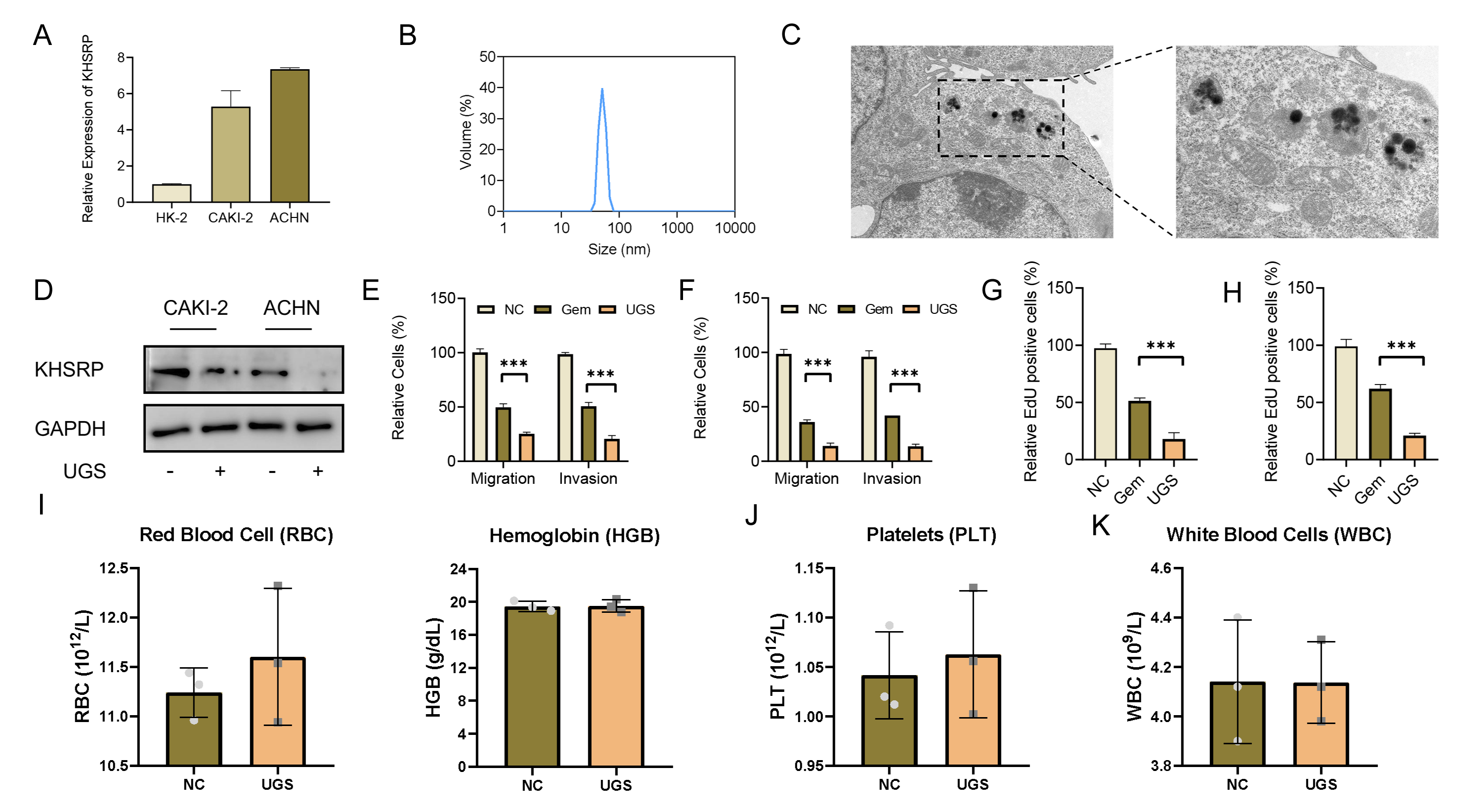

Supplement: Supplementary file 2 [file Image2.TIF]

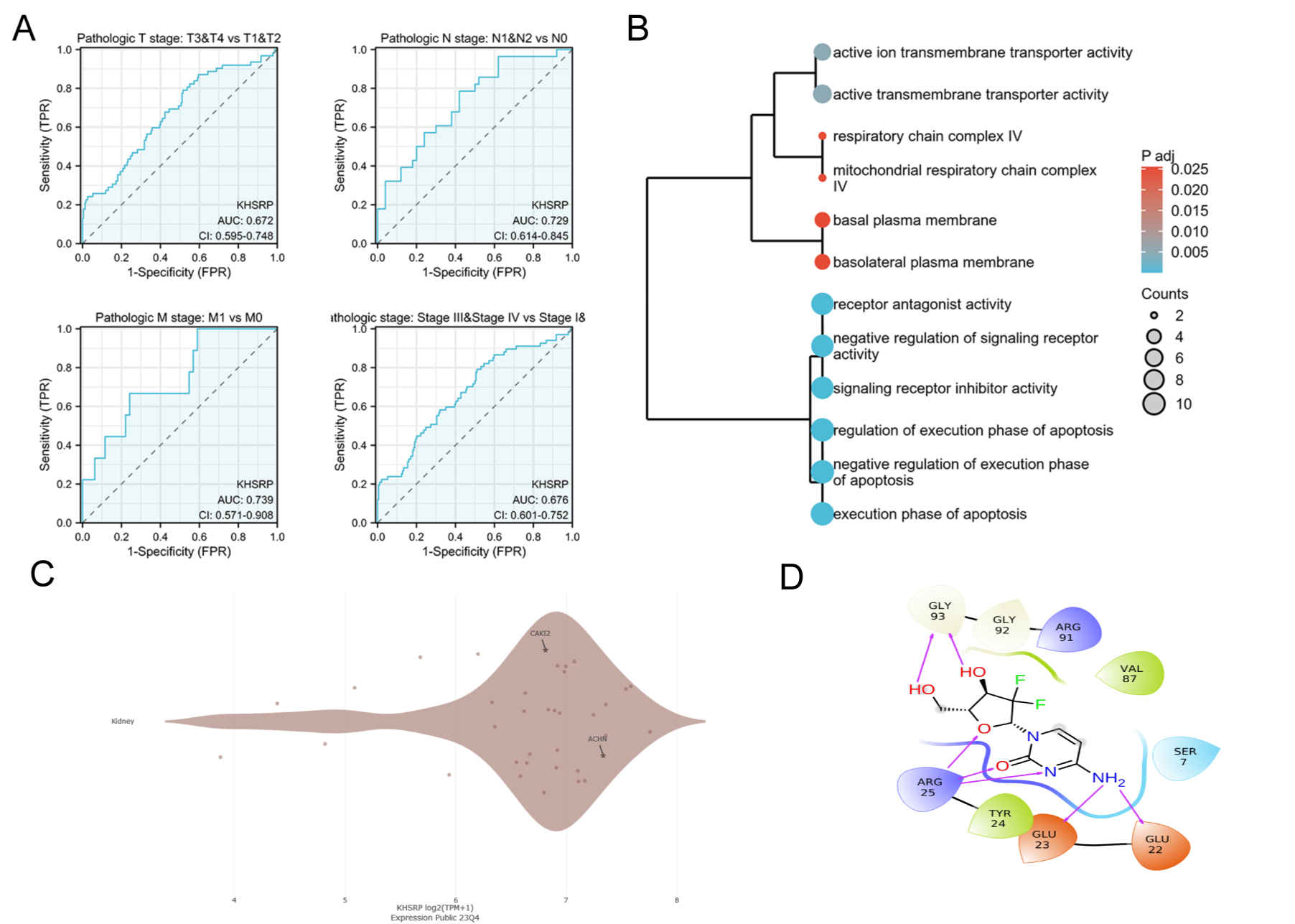

Supplement: Supplementary file 3 [file Image1.TIF]

Figure 1J

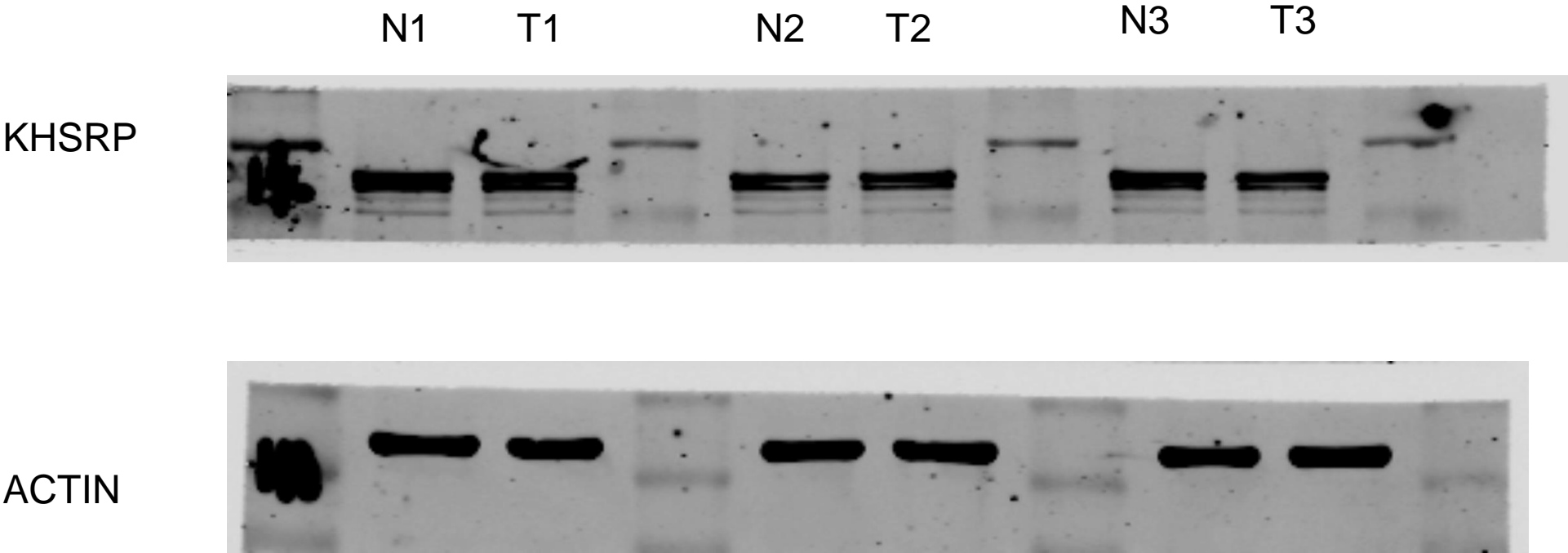

Figure 1k

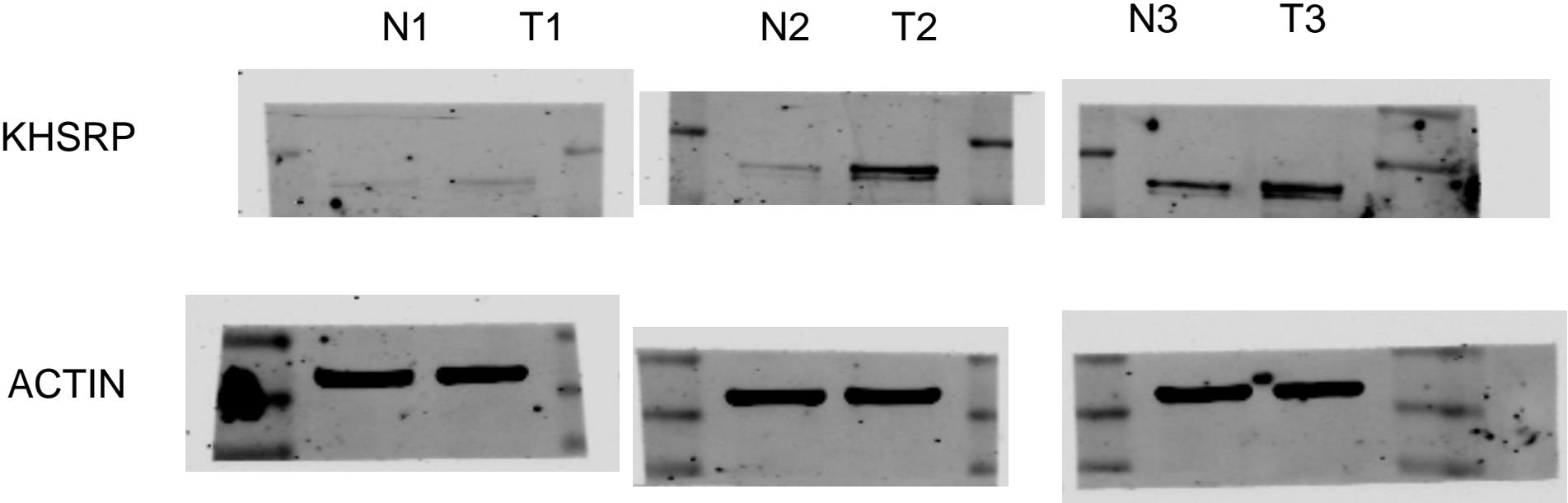

Figure 2D

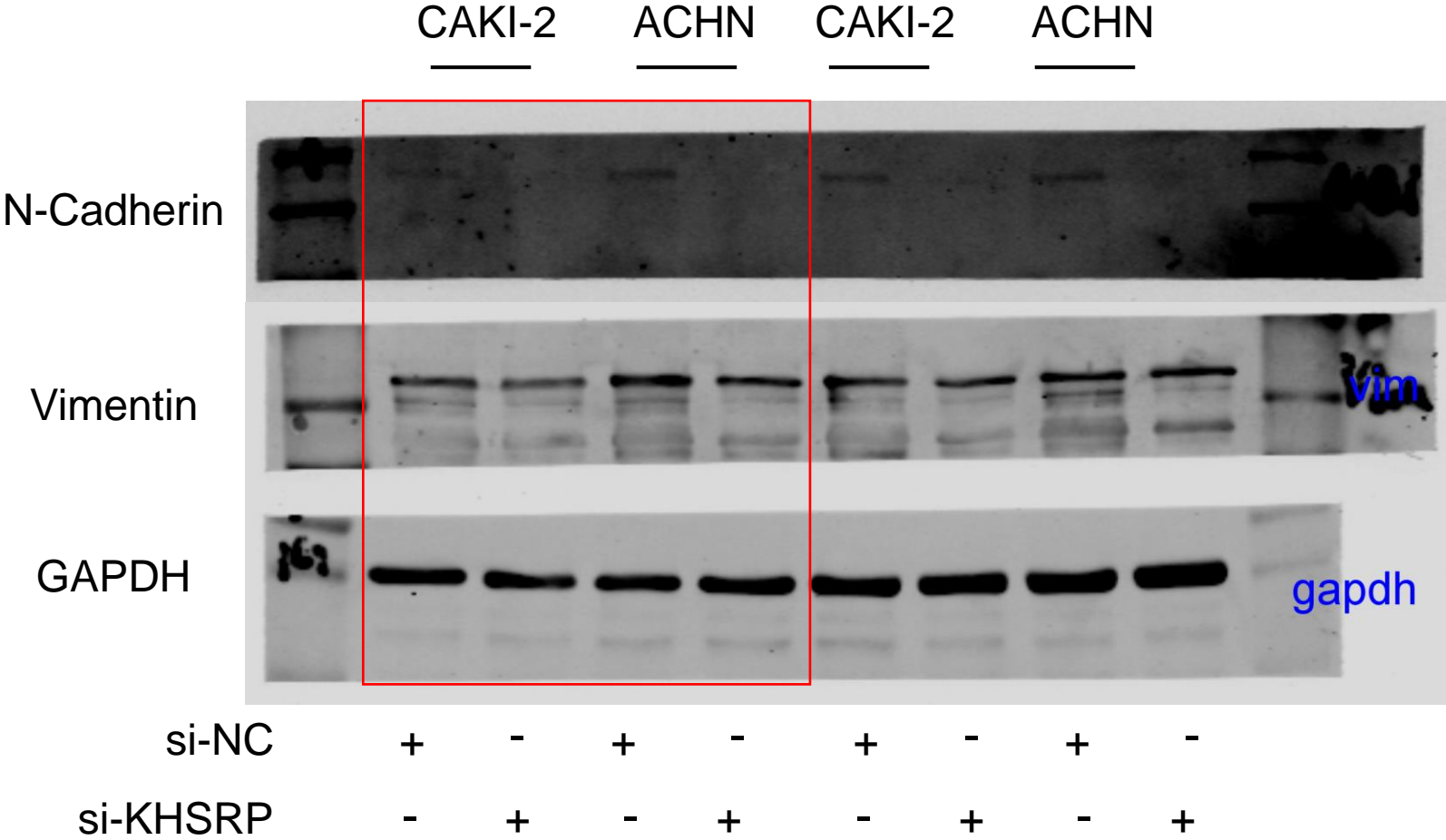

Figure 2F

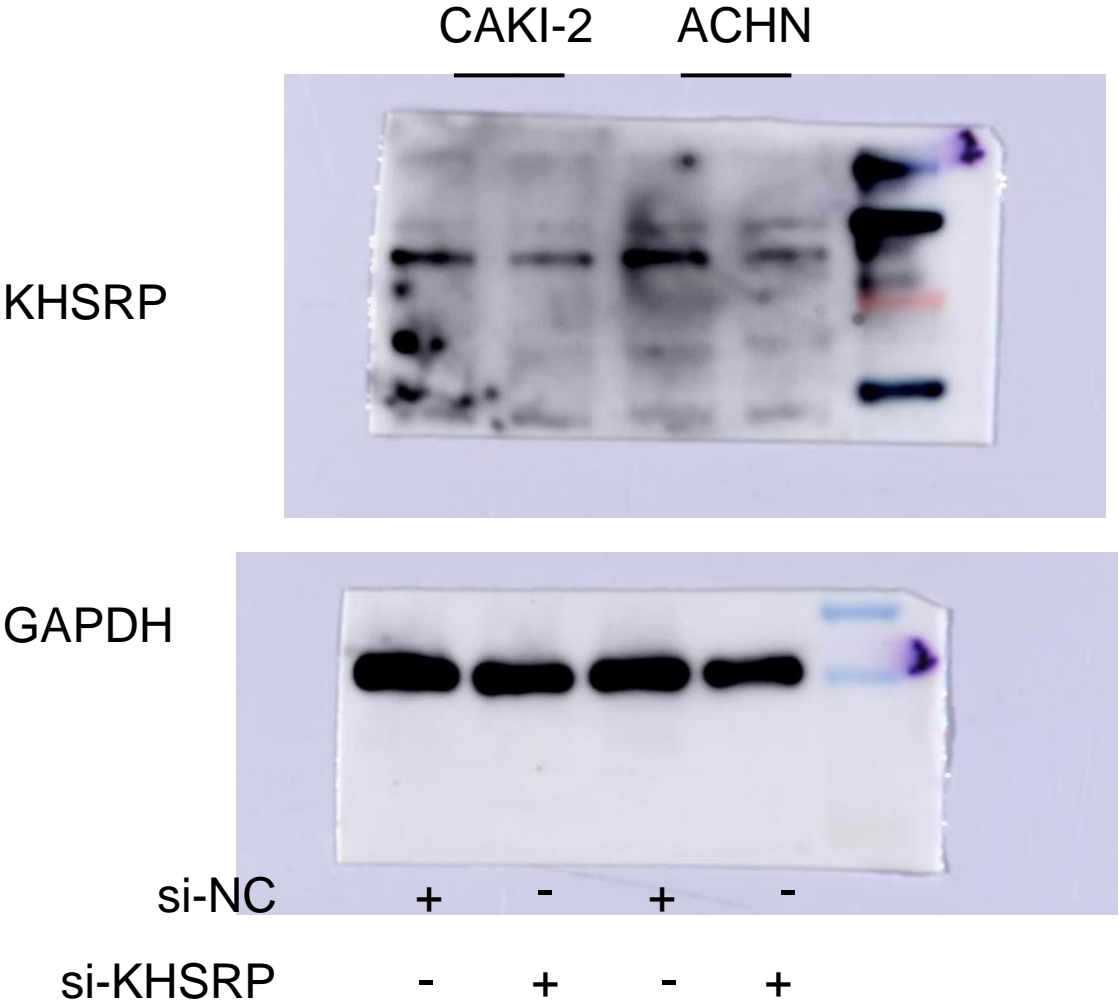

Figure 4D

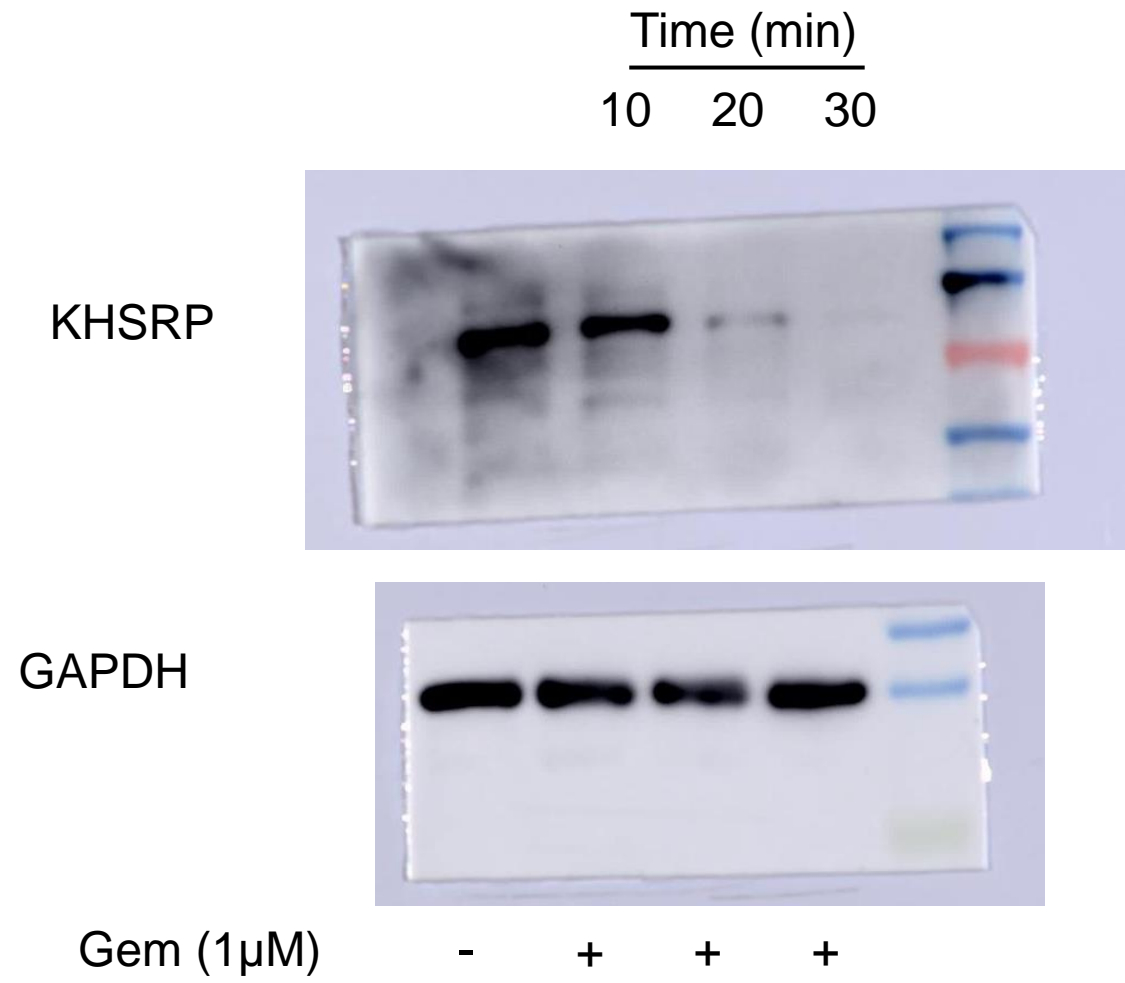

Figure 4O

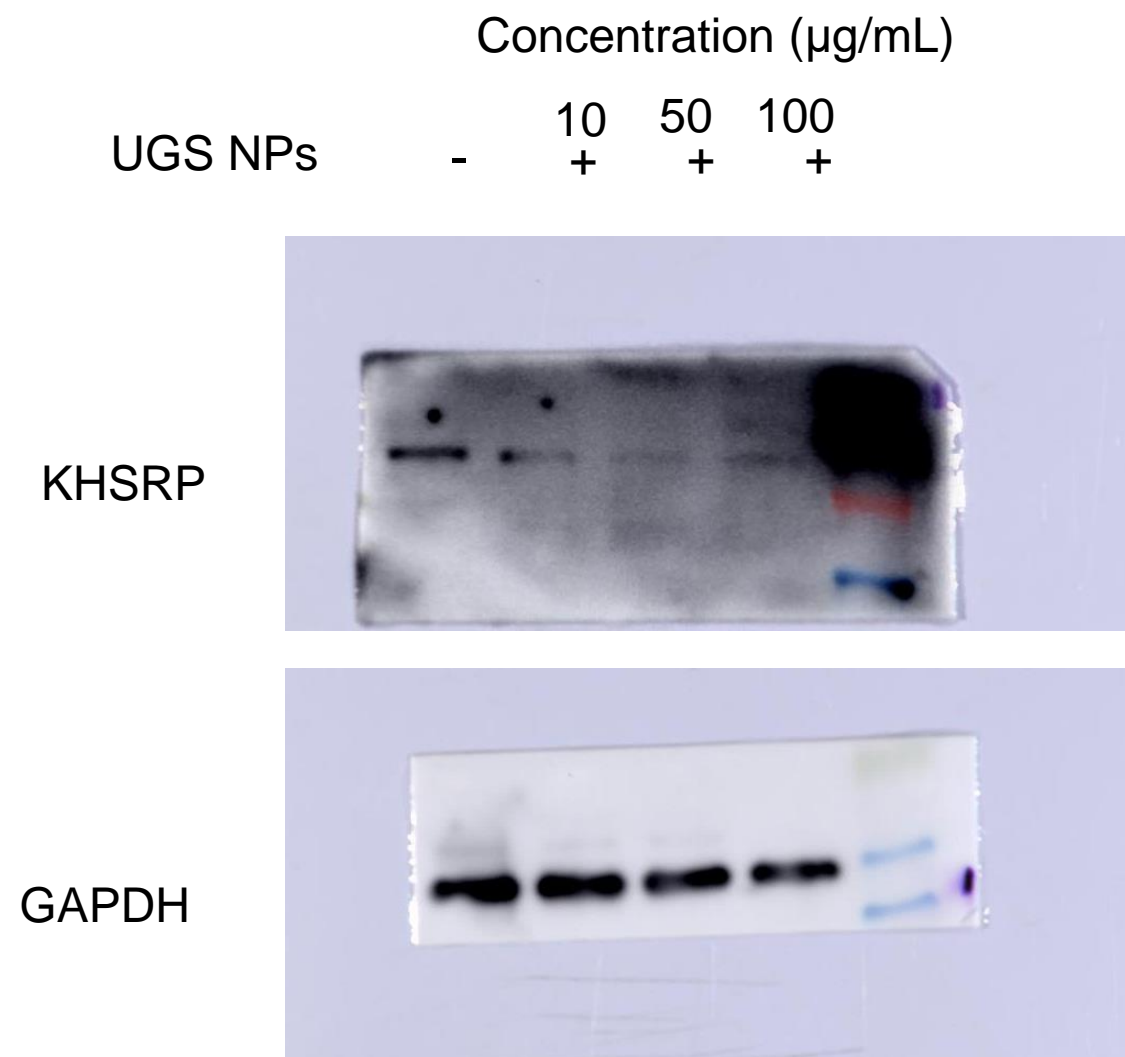

Figure 6H

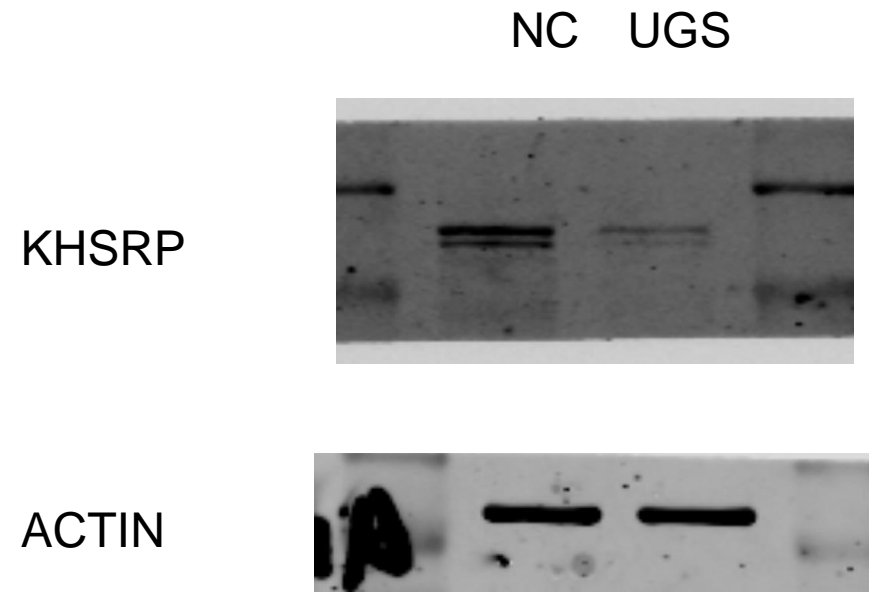

Figure S2B

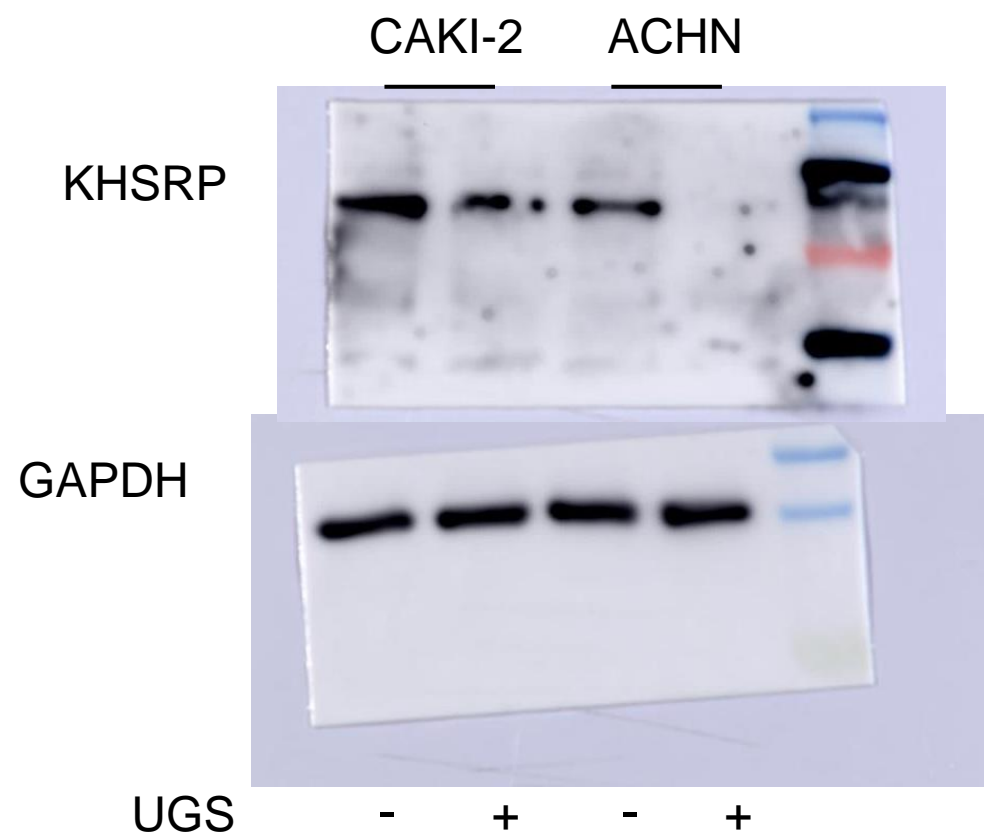

Supplement: Supplementary file 4 [file DataSheet1.pdf]
